# Heterogeneity of germline variants in high risk breast and ovarian cancer susceptibility genes in India

**DOI:** 10.1093/pcmedi/pby010

**Published:** 2018-09-22

**Authors:** Archana Sharma-Oates, Abeer M Shaaban, Ian Tomlinson, Luke Wynne, Jean-Baptiste Cazier, Sudha Sundar

**Affiliations:** 1 Centre for Computational Biology, Haworth Building, University of Birmingham, Edgbaston, Birmingham, UK; 2 Institute of Cancer and Genomic Sciences, College of Medical and Dental Sciences, Haworth Building, University of Birmingham, Edgbaston, Birmingham, UK; 3 Department of Cellular Pathology, University Hospitals Birmingham NHS Foundation Trust, Queen Elizabeth Hospital Birmingham, Mindelsohn Way, Birmingham, UK; 4 Pan-Birmingham Gynaecological Cancer Centre, City Hospital, Dudley Road, Birmingham, West Midlands, UK

**Keywords:** BRCA1, BRCA2, hereditary cancer, sporadic cancer, India, breast cancer, ovarian cancer

## Abstract

Breast and ovarian cancers now account for one in three cancers in Indian women and their incidence is rising. Major differences in the clinical presentation of breast and ovarian cancers exist between India and the United Kingdom. For example, Indian patients with breast cancer typically present a decade earlier than in the UK. Reasons for this could be multifactorial, including differences in underlying biology, environmental risks, and other systematic factors including access to screening. One possible explanation lies in variable incidence or penetrance of germline mutations in genes such as BRCA1 and BRCA2. We performed a methodical database and literature review to investigate the prevalence and spectrum of high-risk cancer susceptibility genes in Indian patients with breast and ovarian cancers. We identified 148 articles, but most studies were small, with inconsistent inclusion criteria and based on heterogeneous technologies, so that mutation frequency could not be reliably ascertained. Data were also often lacking on penetrance, histopathology, and survival outcomes. After filtering out unsuitable studies, only 13 remained, comprising 1028 patients. Large-scale research studies are urgently needed to determine mutation prevalence, spectra, and clinico-pathological features, and hence derive guidelines for screening, treatment, and prevention specific to the Indian population.

## Introduction

The global cancer burden is expected to increase from 14.1 million new cases and 8.2 million deaths in 2012, to 21.7 million cases and 13 million deaths by 2030. However, these large numbers are contrasted by the very diverse nature of cancer that makes every patient unique. Precision medicine has enormous potential to transform cancer care by identifying genomic and epigenetic markers for screening, treatment, and prognosis. These gains are particularly relevant for countries such as India, grappling with both a rising cancer burden and competing demands for essential health care. India’s cancer burden, currently estimated at over 1.5 million new cases is predicted to nearly double in the next 20 years, with age-adjusted mortality rates of 64.5 per 100 000 (GLOBOCAN 2012).^1^ Cumulatively, breast, cervical, ovarian, and uterine cancers account for more than 70% of cancers in women in India, thus establishing tackling women’s cancers as high priority for healthcare providers and research.^2^

**Figure 1.**
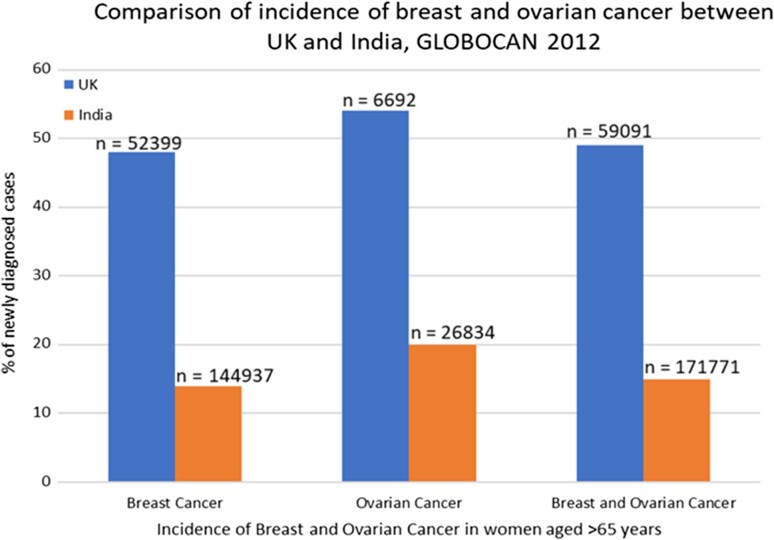
Comparisons between UK and India by age of newly diagnosed BOC incidence in women.^3^

Significant phenotypic differences exist in breast and ovarian cancers between patients in India and in the UK. The incidence of breast and ovarian cancer is relatively low in India in comparison with the UK: breast cancer 23.8 versus 92.9 cases per 100 000 women in the UK, ovarian cancer 4.9 versus 11.7/100 000 women in the UK (GLOBOCAN 2012).^1^ However, a high proportion (~11-26%) of Indian patients with breast cancer present at ages younger than 35 years.^3^ Conversely, approximately half of newly diagnosed breast and ovarian cancer cases occur in women aged 65 years and older in the UK, compared with only 15% in India (Fig. 1). The incidence of the more aggressive histological type of breast cancer, triple-negative disease, is also estimated to be higher at 31% in India, nearly double that of the UK.^5^ Breast cancer incidence also fluctuates substantially across India, with age-standardised incidence rates varying between 41/100 000 rate in urban centres such as New Delhi and 12.4/100 000 in rural cancer registries, thus adding a further layer of complexity.^6^

These phenotypic differences could be a result of differences in tumour biology such as differences in the incidence of high-risk germline susceptibility genes, environmental modifiers,^7,8^ or systematic factors such as access to screening and treatment. Germline mutations in high-risk susceptibility genes (e.g. BRCA1, BRCA2) account for 5-10% of breast cancers and up to 20% of ovarian cancers in white Europeans.^9–12^ Women with a germline BRCA1 mutation have a lifetime risk of ovarian cancer by age 70 years of up to 63% and of breast cancer by age 70 years of up to 85%.^13^ Risks of ovarian and breast cancers in women by age 70 years among BRCA2 carriers are reported to be up to 27% and 84%, respectively. Other genes in which germline mutations confer susceptibility to breast and/or ovarian cancer, albeit with lower frequency and penetrance include PALB2, TP53, PTEN, CDH1, STK11, CHEK2, RAD51, and ATM.^14^

We systematically reviewed the literature and relevant data repositories to characterise the prevalence and spectrum of germline variants in breast and ovarian cancer susceptibility genes in the Indian population, including putative BRCA1 and BRCA2 founder mutations. We excluded SNPs with high frequency in the population. We investigated the literature for details of clinical, family history, pathology, and survival data in these patients.

## Methods

### Search strategy, inclusion and exclusion criteria

A comprehensive literature search was performed to include articles published between 1 January 1990 and 1 December 2016 using the following search terms on ethnicity, condition, and high penetrance genes (Table 1): ‘India and (breast cancer or ovarian cancer) and (BRCA1 or BRCA2 or PALB2 or TP53 or PTEN or CDH1 or STK11 or CHEK2 or RAD51C or RAD51D or ATM or BARD1 or NBN or MLH1 or MSH2 or MSH6 or PMS2 or EPCAM)’ in EMBASE and PubMed/Medline to identify relevant published and unpublished studies as well as studies in progress. Further searches were carried out in the BIC^15^ database using the keyword ‘Indian’ in the ethnicity fields and also in the ClinVar database.^16^ Additional database searches included the 1000genomes^16^, TCGA,^13^ COSMIC^18^, dbSNP^19^, ICGC^20^, HGMD^21^, ExAC^22^, and the GWAS catalog^23^.

**Table 1.** List of genes, with high and moderate penetrance, used in the search terms in association with breast and ovarian cancer as well as Lynch syndrome.

High-penetrance genes	Moderate-penetrance genes	Lynch syndrome genes
BRCA1	CHEK2	MLH1
BRCA2	RAD51C	MSH2
PALB2	RAD51D	MSH6
TP53	ATM	PMS2
PTEN	BARD1	EPCAM
CDH1	NBN	
STK11		

This initial search was supplemented by checking reference lists, and contact with authors of included studies for information on any relevant published or unpublished studies. No language restrictions were applied. Two reviewers assessed titles, abstracts, and keywords to select potentially relevant studies from the retrieved list of articles.

### Study selection criteria for literature search

All studies included in the analysis met the following inclusion criteria: (i) data reported on any genes included in Table 1; (ii) at least 10 patients of Indian origin; and (iii) contained DNA sequence variation data. The susceptibility genes selected are those commonly tested in clinical practice. Lynch syndrome genes were included as they confer susceptibility to ovarian cancer in addition to colon and uterine cancers (Table 1). Importantly, inclusion was not restricted by NCCN or Manchester definitions of familial risk to ensure broad inclusion of studies with available data.

**Figure 2.**
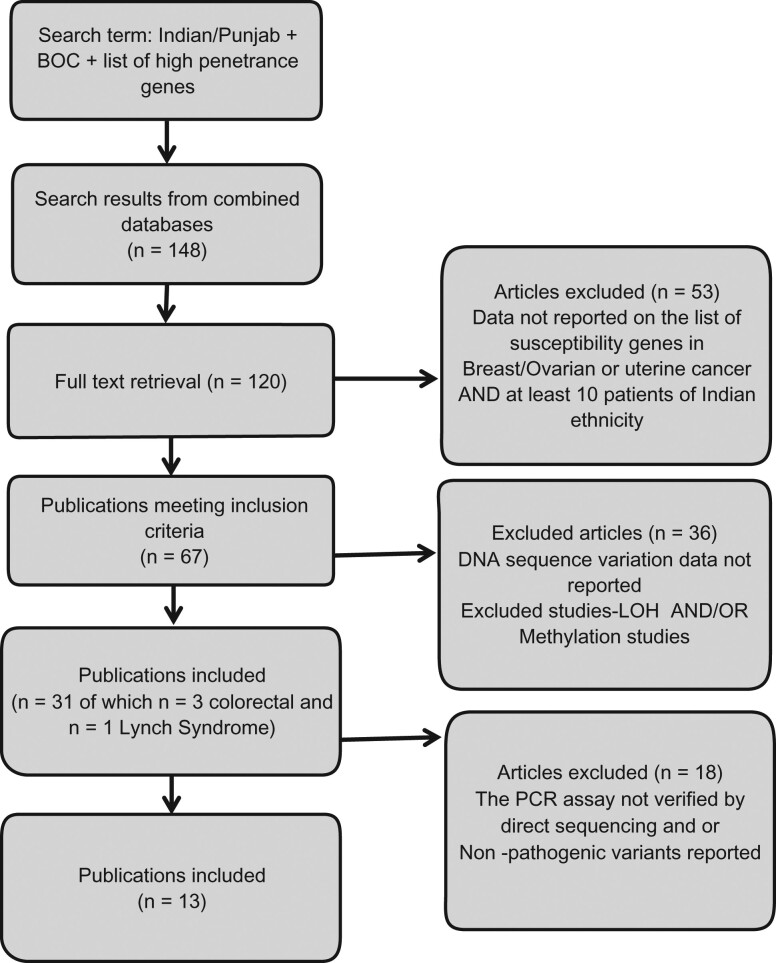
Flow diagram illustrating the criteria for selection of publications and corresponding number of articles.

The exclusion criteria were: (i) articles containing data limited to loss of heterozygosity and/or methylation studies; (ii) duplicate publications; (iii) studies that did not perform direct DNA sequencing to validate variants detected by PCR-based techniques using re-amplified genomic DNA; and (iv) studies that did not screen the entire susceptibility gene. If studies had overlapping data, only the latest or largest study was included (Fig. 2).

The first step of a two-stage selection process involved screening titles and abstracts. Subsequently, for all references categorised as ‘include’ or ‘uncertain’ by both reviewers, full text was retrieved wherever possible and final inclusion decisions were made on the full paper. Data extraction was carried out using pre-designed and piloted data extraction forms with differences resolved by consensus and/or arbitration involving a third reviewer.

### Data extraction from literature search

Three reviewers extracted detailed information relating to variants; clinical evidence, including family history when available; clinical diagnosis; and histopathology. The information collected included the following: year of publication; authors’ names; journal; geographic location of study; cancer type; genotyping methods; details of germline variant, total numbers of cases and controls; frequencies of variant carriers in cases and controls; histopathology; overall and progression-free survival where available; and age of presentation.

All variants extracted from the publications were queried against the BIC database for BRCA1 and BRCA2 genes and ClinVar^16^ to confirm whether they had been reported previously by other studies and to obtain their pathogenic classification. The SNP identifier for each of the variants, where available, was obtained from the dbSNP database.^24^

## Results of literature search

### Characteristics of included studies

The combined search for key terms led to the selection of 148 articles. After screening titles, abstract, and keywords, we extracted 120 full texts of articles considered eligible for inclusion. After reviewing the full texts and citations, we identified 67 studies meeting the inclusion criteria of which 31 contained data suitable for extraction. Of the 31 articles, only 13 articles contained usable data that satisfied both the inclusion and exclusion criteria (Fig. 2, Table 2). These publications included familial breast and/or ovarian cancer as well as sporadic cases. For the purposes of this review, we used a broad definition of FEOTN (familial/early-onset/triple-negative) based on the studies included in the review, specifically one or more of the following: at least one first-degree relative with breast and/or ovarian cancer irrespective of age; early onset breast and/or ovarian cancer diagnosed with a family history; relatives affected first or second degree; triple-negative breast cancer in an early onset case; or bilateral breast cancer diagnosed < 50 years. Data were included from probands and from family members who were carriers, where given. We also included data from sporadic cancer patients where the paper contained this information. However, none of the publications on sporadic cases reviewed reported any pathogenic germline variants and therefore we focused our analysis on FEOTN cases (Fig. 2).

**Table 2.** Publications reporting variations in high-penetrance breast and ovarian cancer genes.

Year	Geographic location	Number cases	Number of controls	Cancer subtype	Gene names	Method	Title	Journal
2009	South India	61	100	Breast cancer	BRCA1 and BRCA2	Heteroduplex analysis using CSGE and direct sequencing	BRCA1 and BRCA2 germline mutation analysis among Indian women from south India: Identification of four novel mutations and high-frequency occurrence of 185delAG mutation	*J Biosci*;**34**:415
2002	North India	20	50	Breast cancer	BRCA1 and BRCA2	Heteroduplex analysis/USB PCR- products sequencing kit	BRCA1 and BRCA2 in Indian patients with breast cancer	*Hum Mutat*;**20**:473–74
2006	Srinagar, Jammu, and Kasmir, India	63	63	Breast cancer	BRCA1 and TP53	PCR-SSCP (single stranded conformational polymorphism) followed by direct sequencing	BRCA1 and TP53 mutation spectrum of breast carcinoma in an ethnic population of Kashmir, an emerging high-risk area	*Cancer Letters*;**248**:308–20
2003	North India, New Delhi	40	50	Breast cancer	BRCA1	SSCP and direct sequencing	BRCA1 germline mutations in Indian familial breast cancer	*Hum Mutat*;**21**:98–9
2012	Mumbai	151	50	Breast cancer	BRCA1 and BRCA2	PCR+direct sequencing	BRCA1/BRCA2 gene mutations/SNPs and BRCA1 haplotypes in early-onset breast cancer patients of Indian ethnicity	*Med Oncol*;**29**:3272-81. doi: 10.1007/s12032-012-0294-9. Epub 2012 Jul 3
2006	New Delhi, Northern India	204	140	Breast cancer	BRCA1 and BRCA2	Heteroduplex analysis of PCR amplicons using exon specific primers	Contribution of germline BRCA1 and BRCA2 sequence alterations to breast cancer in Northern India	*BMC Med Genet*;**7**:75
2016	56/141 from North India, 63 from South India	141	250	Breast and ovarian cancer	BRCA1, BRCA2, ATM, BRIP1, CDH1, CHEK2, NBN, PALB2, PTEN,RAD51C, RAD51D, STK11, and TP53	Illumina MiSeq and sanger sequencing and MLPA (multiplex ligation-dependant probe amplification)	Detection of high frequency of mutations in a breast and/or ovarian cancer cohort: implications of embracing a multi-gene panel in molecular diagnosis in India	*J Hum Genet*;**61**:515–22. doi: 10.1038/jhg.2016.4. Epub 2016 Feb 25
2008	Indian ethnicity, Malaysia	22	?	Breast cancer	BRCA1 and BRCA2	DHPLC and DNA sequencing	Evaluation of BRCA1 and BRCA2 mutations and risk-prediction models in a typical Asian country (Malaysia) with a relatively low incidence of breast cancer	*Breast Cancer Res*;**10**:R59. doi: 10.1186/bcr2118. Epub 2008 Jul 16
2002	Trivandrum, South India	14	?	Breast and ovarian cancer	BRCA1	Conformation sensitive gel electrophoresis and direct sequencing of PCR products	Germline BRCA1 mutation analysis in Indian breast/ovarian cancer families	*Cancer Biol Ther*;**1**:18–21
2007	Kerala, South India	102	?	Breast and ovarian cancer	BRCA2	Direct sequencing	Novel germline mutations in BRCA2 gene among 96 hereditary breast and breast–ovarian cancer families from Kerala, South India	*J Cancer Res Clin Oncol*;**133**:867–74
2004	New Delhi	65	69	Breast and ovarian cancer	BRCA1 and BRCA2	Direct sequencing	Novel germline mutations in the BRCA1 and BRCA2 genes in Indian breast and breast–ovarian cancer families	*Hum Mutat*;**23**:205
2014	Indian ethnicity, Malaysia	54	?	Breast cancer	BRCA1 and BRCA2	PCR and sanger sequencing	Recurrent mutation testing of BRCA1 and BRCA2 in Asian breast cancer patients identify carriers in those with presumed low risk by family history	*Breast Cancer Res Treat*;**144**:635–42. doi: 10.1007/s10549-014-2894-x. Epub 2014 Mar 1
2015	Chennai, South India	91	2	Breast and ovarian cancer	BRCA1, BRCA2, TP53, RAD50, RAD52, ATM, and TP53BP1	Illumina HiScanSQ system and sanger sequencing and PCR-dHPLC	Targeted resequencing of 30 genes improves the detection of deleterious mutations in South Indian women with breast and/or ovarian cancers	*Asian Pac J Cancer Prev*;**16**:5211–7

**Figure 3.**
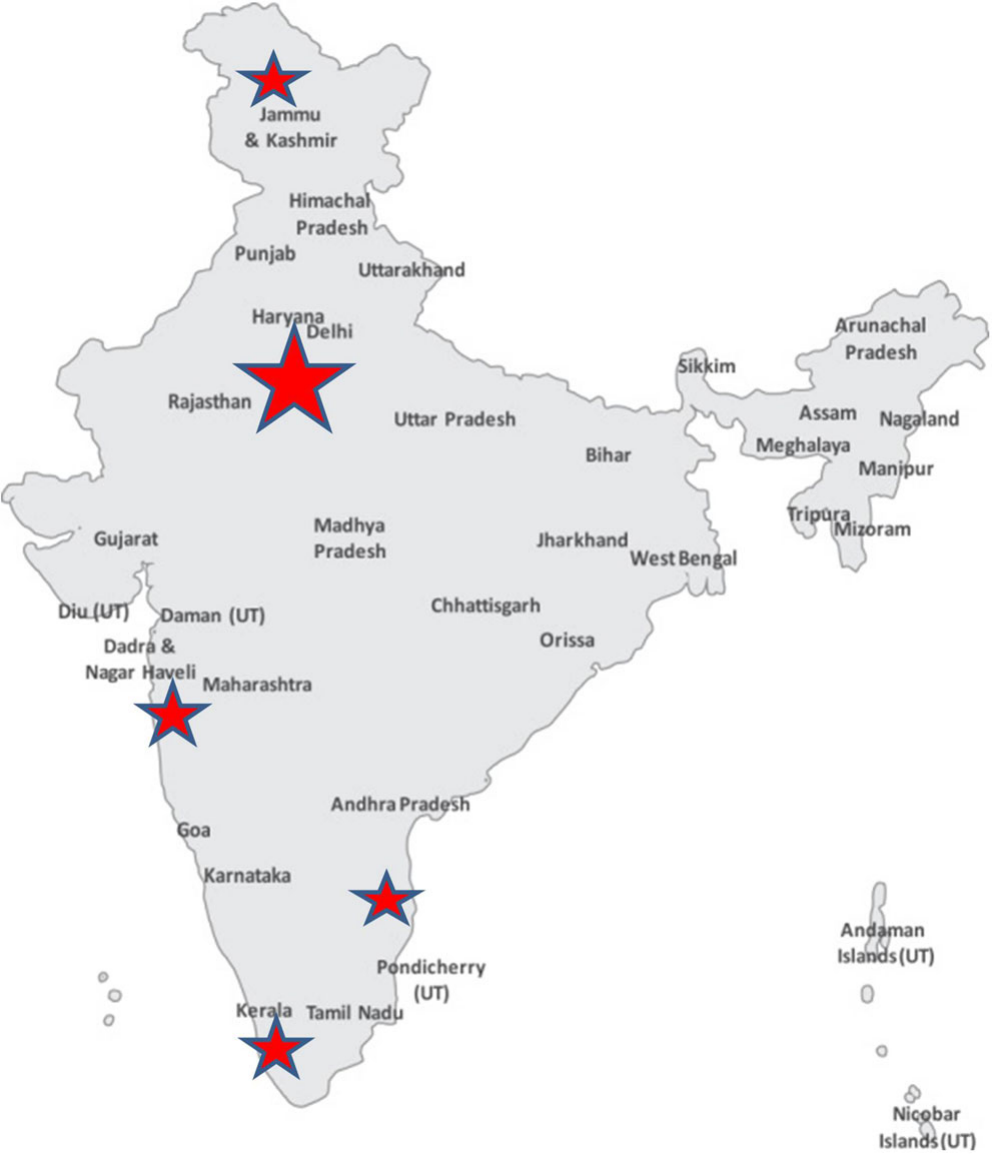
Geographical distribution of the cohorts from the selected studies. The size of the stars are proportional to the size of the study cohort.

We identified a total of 1028 breast and/or ovarian cancer cases from the 13 studies. A breakdown of the number of studies from different categories of breast and/or ovarian cancer is presented in Table 3. The majority of the studies were conducted in or near the largest cities of India with the exception of two that were carried out within the Indian populations of Malaysia and Singapore. The patients recruited in any study usually resided in or near the big cities, which are densely populated and are more affluent than the rural populations of India (Fig. 3).

**Table 3.** Breakdown of cancer subtypes from data extracted.

Type of cancer	Category	Total number of cases	Number of studies
Breast cancer	Familial	529	12
	Early onset	218	6
	Sporadic	128	5
	Uncategorised	105	2
Ovarian cancer		14	2
Breast and ovarian cancer		29	3

### Platforms used for genetic testing

Many different platforms were used for genetic testing in the 13 studies, with the majority using PCR-based approaches including hetero-duplex formation, single-strand conformation polymorphism (SSCP) analysis, denaturing high-performance liquid chromatography (dHPLC), and Sanger sequencing.

Only two studies with a cohort size of 141 and 91 used next generation sequencing (NGS) with Illumina HiScanSQ system, and these also reported the highest proportions of variants in the cohort.

### Study findings on prevalence of cancer susceptibility genes

All 13 FEOTN publications reported data on BRCA1 and/or BRCA2 and only three studies tested for other susceptibility genes such as TP53, RAD50, RAD52, ATM, and CHEK2, with mutations in these found very rarely if at all. We therefore limited our analysis to BRCA1 and BRCA2 genes. Twelve studies reported previously identified pathogenic BRCA1 variants and 10 reported novel variants they considered likely to be pathogenic. The novel variants were not present in any of the online databases listed in the Methods section. Initially, we considered variants causing protein truncation only to be likely pathogenic. We then predicted the functional effects of non synonymous missense variants using SIFT, PolyPhen and CADD and identified 2 additional variants, 5360A>C and 5377G>A, considered deleterious/probably damaging by all three algorithms (Supplementary Table 1). In total, we identified 26 previously reported pathogenic variants and 18 novel likely pathogenic variants for BRCA1 from a total cohort of 926 (Tables 4 and 5). In combination, the previously reported and the novel variants were detected in 71/926 cases, 39 of whom carried the ‘Ashkenazi’ 185delAG mutation.

**Table 4.** Previously reported pathogenic BRCA1 variants identified from the literature search that are also present in BIC and ClinVar.

		HGVS annotation			BIC		Clinical significance					BIC entries
BRCA1	EXON/Intron	cDNA	Protein	Variant type	Designation	Class	ClinVar classification	dbSNP id	Number of studies reporting variant	Total cases (does not include controls)	Carrier	Number of cases
	2	c.66_67delAG	p.Leu22_Glu23LeuValfs	F	*185delAG	5	Pathogenic	rs80357713	10	927	39	2038
	20	c.5260G>T	p.Glu1754Ter	N	E1754X	5	Pathogenic	rs80357432	1	40	1	20
	11	c.2864C>A	p.Ser955Ter	N	S955X	5	Pathogenic	rs80357295	1	61	1	4
	11	4213delT	Leu.1365->Stop	N	-	-	Pathogenic	rs398122681	1	61	1	-
	18	5267T->G	p.Tyr1716Ter	N	-	-	Pathogenic	rs397509230	1	61	1	-
	11	3450delCAAG/c.3331_3334delCAAG	p.Gln1111_Glu1112?fs	F	3450del4	5	Pathogenic	rs80357903	1	61	1	43
	5	c.212+1G>T	-	SS	IVS5+1G>T	-	Pathogenic	rs80358042	1	20	1	6
	20	c.5241delA	p.Gln1747 = fs	F	5360delA	5	Pathogenic	rs80357791	1	40	1	1
	13	c.4327C>T	p.Arg1443ter	N	R1443X	5	Pathogenic	rs41293455	1	22	1	131
	12	c.4183C>T	p.Gln1395Ter	N	Q1395X	5	Pathogenic	rs80357260	1	124	1	28
	11	c.671-1G>T	-	IVS	IVS10-1G>T	Pending	Pathogenic	rs80358020	1	91	1	1
	11	c.5074+1G>A	-	SS	IVS17+1G>A	Pending	Pathogenic	rs80358053	1	91	1	3
	11	c.3553G>T	p.Glu1185Stop	F	-	-	Pathogenic	rs397509081	1	43	1	-
	11D	c.4065_4068delTCAA	p.Asn1355_Gln1356?fs	F	4184del4	5	Pathogenic	rs80357508	1	204	1	144
	11D	3596del4/c.3477_3480delAAAG	p.Ile1159Metfs	F	3596del4	5	Pathogenic	rs80357781	1	204	1	3
	15/14	c.4485-1G>A	-	IVS	IVS14-1G>A	Pending	Pathogenic	rs80358189	1	151	3	2
	11	c.2275C>T	p.Gln759ter	N	Q759X	5	Pathogenic	rs80356999	1	151	2	1
	11	c.2338C>T	p.Gln780ter	N	Q780X	5	Pathogenic	rs80356945	1	151	2	36
	11	c.3607C>T	Arg1203ter	N	R1203X	5	Pathogenic	rs62625308	1	151	1	36
	3	235G>A/c.116G>A	Cys39Tyr	M	C39Y	Pending	Conflicting interpretations of pathogenicity, not provided. Pathogenic (4);Uncertain significance (1)	rs80357498	1	151	1	5
	5	c.182G>A	Cys61Tyr	M	C61Y	Pending	Conflicting interpretations of pathogenicity, Pathogenic (3);Uncertain significance (1)	rs80357093	1	151	1	6
	10	c.3352C>T	p.Gln1118Ter	NS	-		Pathogenic	rs397507215	1	141	2	-
	15	c.4837_4838delAGinsGCC	p.Ser1613Alafs	Indel	-		Pathogenic	rs730880287	1	141	2	-
	16	c.5035delC	p.Leu1679Terfs	Indel	-		Pathogenic	rs80357896	1	141	1	-
	20	c.5251C>T	p.Arg1751Ter	N	-		Pathogenic	rs80357123	1	22	1	-
	11	1173G>T	p.Glu352Ter	N	E352X		Pathogenic	rs80357472	1	22	1	-
	2	180delA	Stop22	F	180delA		Pathogenic	rs273902778	1	22	1	3

N = Nonsense, F = frameshift, SS = splice site, IVS = Intervening sequence ie. the intron, Indel = insertion and deletion. Recurrent variant detected in multiple studies: Vaidyanathan *et al.* (61 cases, 10 carriers of 185delAG), Saxena *et al.* (204 cases, 1 carrier of 185delAG), Mannan *et al.* (141 cases, 6 carriers of 185delAG), Kumar *et al.* (14 cases, 1 carrier of 185delAG), Hedau *et al.* (124 cases, 2 carriers of 185delAG), Kang *et al.* (54 cases, 4 carriers of 185delAG), Rajkumar *et al.* (91 cases, 10 carriers of 185delAG), Juwle *et al.* (151 cases, 2 carriers of 185delAG), Thirthagiri *et al.* (65 cases, 2 carriers of 185delAG), Valarmathi *et al.* (65 cases, 2 carriers of 185delAG). Total: 927 cases, 39 carriers of 185delAG.

**Table 5.** Novel likely pathogenic BRCA1 variants.

BRCA1	Exon/intron	HGVS annotation cDNA	Protein	Variant type	Number of studies contributing to the total number of cases	Total cases (does not include controls)	Carrier
	2	c.3672G>T	p.Glu1185Stop	N	1	65	1
	7	c.512dupT	p.Gln172ThrfsTer10	Indel	1	141	1
	10	c.779dupA	p.Tyr261ValfsTer1	Indel	1	141	1
	10	c.1155G>A	p.Trp385Ter	NS	1	141	1
	10	c.1416delC	p.Asn473ThrfsTer2	Indel	1	141	1
	12	c.4349C4A	p.Ser1450Ter	F	1	141	1
	22	c. 5440dupG	p. Ala1814GlyfsTer16	SS	1	141	1
	16	4956insG	TGA at codon	F	1	124	1
	11	4213delT	Leu.1365->Stop	N	1	61	1
	18	5267T->G	p.Tyr1716Ter	N	1	61	1
	11	1027delA	delA-ter313 (codon303)	F	1	14	1
	16	4956insG/c.4183C>T	p.Gln1395Ter	F	1	124	1
	20	5339G>T>G	p.Glu754Ter	M	1	40	1
	11	3867G>T	p.Glu250Ter	N	1	40	1
	5	(nucleotide) 295delCA	Translation stop at codon 64	F	1	61	1
	11	1052delT	Stop313	F	1	151	2
	8	632insT	Stop181	F	1	151	4

N = Nonsense, F = frameshift, SS = splice site, IVS = Intervening sequence ie. the intron, Indel = insertion and deletion.

For BRCA1, there were seven additional recurrent mutations, five in BIC and/or ClinVar and two that were novel (Tables 4 and 5). Of the five previously reported variants, c.2275C>T, c.2338C>T, c.3352C>T, and 4838delAGinsGCC each occurred in two cases and the other, c.4485-1G>A, occurred in three cases. The two novel variants were c.1052delT and c.632insT, the former detected in four cases and the latter in two cases, all from single studies (Table 5).

For BRCA2, there were four variants previously reported as pathogenic in ClinVar detected in the FEOTN cases; these were detected in 6/974 cases. The only recurrent variant, 6079del4, was detected in 3/974 cases from two different studies (Table 6). The number of variants reported to be novel and likely pathogenic was 16, and each of these variants was detected in single cases in single studies (Table 7). Furthermore, there were 9 non synonymous missense variants of which only one,c.3578T>C, was considered deleterious/probably damaging by SIFT, POlyphen and CADD (Supplementary Table 2).

**Table 6.** Previously reported pathogenic BRCA2 variants identified from the literature search that are also present in BIC and ClinVar.

EXON/Intron	HGVS annotation		Variant type	BIC		ClinVar	dbSNP	Number of studies	Total cases	Control	Carrier	Number of entries in BIC
	cDNA	Protein		Designation	Class	Clinical significance						
11	c.5851_5854delAGTT	p.Ser1951_Leu1952?fs	F	6079del4^a^	5	Pathogenic	rs80359544	2	212	150	3	11
21	c.8754+1G>A	p.Leu1198Ter	SS	-	-	pathogenic	rs397508006	1	22	?	1	-
11	c.3847_3848delGT	p.Val1283Lysfs	F	4075delGT	5	Pathogenic	rs80359405	1	151	50	1	64
22	c.8869C>T	p.Gln2957Ter	N	Q2957X	5	Pathogenic	rs276174913	1	22	0	1	1
^a^Identified in two different studies												
11	c.5851_5854delAGTT	p.Ser1951_Leu1952?fs	F	6079del4^a^	5	Pathogenic	rs80359544	1	151	50	2	11
11	c.5851_5854delAGTT	p.Ser1951_Leu1952?fs	F	6079del4^a^	5	Pathogenic	rs80359544	1	61	100	1	11

^a^Recurrent variant detected in multiple studies. N = Nonsense, F = frameshift and SS = splice site.

**Table 7.** Novel likely pathogenic BRCA2 variants.

BRCA2	EXON/Intron	HGVS annotation cDNA	Protein	Variant type	Number of studies contributing to the total number of cases	Total cases (does not include controls)	Carrier
	11	c.5076delAA	stop1617	F		151	1
	25	c.9608G>A	p.Trp3127Ter	N		151	1
	11	63761insAA	Stop 2051	F	1	204	1
	19	c.85761nsC	Stop 2797	F	1	204	1
	27	9999delA	Stop3275	F	1	204	1
	11	c.3187C>T	p.Gln1063Ter	NS		141	1
	11	c.3186_3189delTCAG	p.Ser1064LeufsTer12	Indel		141	1
	11	c.4642delAA	Stop1480	F		102	1
	11	c.4926insGACCC	Stop1575	F		102	1
	11	c.5227dupT	Stop1676		1	65	1
	11	c.5242dupT	Stop1676		1	65	1
	11	c.6180dupA	Stop2002		1	65	1
	22	nt 9097	Gln2957	F, N and SS	1	22	1
	11	4866InsT	Asp1547Ter	FS	1	61	1
	11	c.4642delAA	Stop1480	F	1	102	1
	11	c.4926insGACCC	Stop1575	F	1	102	1

N = Nonsense, F = frameshift, SS = splice site, IVS = Intervening sequence ie. the intron, Indel = insertion and deletion.

### Prevalence of founder mutations in BRCA1 and BRCA2

Ten of the 13 studies reported data on the putative founder mutation BRCA1 185delAG (Fig. S1, see online supplementary material). The mutation was detected in 39/927 (4.2%) cases with breast or ovarian cancer, the majority being from South India or Malaysians of Indian descent. The frequency of 185delAG varied, for example one study from New Delhi found only one carrier in 204 cases, but a high prevalence was reported in Bangalore (10/61 cases, 0/100 controls, Fisher exact test *P* = 3.7×10^-5^) and Chennai (10/91 cases, 0/2 controls)^25,26^ (Table 2).

The reported BRCA2 founder mutation 6174delT was not detected in any of the studies included in our analysis.^2^ Frequencies of BRCA mutations identified in the included studies in the Indian population are contrasted with those of white European populations (Tables 4 and 6).

### BIC and ClinVar search and additional database search for variants from Indian ethnicity cases

The BIC and the ClinVar databases contain DNA sequence variations reported by genetics clinics from across the world. The majority of the DNA variants in these repositories are unpublished. The most frequent reported entry in BIC for the BRCA1 gene was 185delAG, which was also the most prevalent in our analysis (Table 8). Eight out of the 20 top entries in BIC were also detected in our literature survey, although not all of these variants were shown to be pathogenic (Table 8). None of the pathogenic BRCA2 variants identified from our literature search were present in the top 20 BIC entries for BRCA2 (Table 9).

**Table 8.** Top 20 BIC entries for BRCA1.

	BIC designation	Number of entries in BIC	Number of studies	Total cases (excluding controls)	Number of carriers	Pathogenicity
1	**185delAG**	2038	10	840	39	Pathogenic
2	**5382insC**	1093	1	92	7	Pathogenic
3	4427T>C	251				
4	**S1613G**	248	2	226	2	Benign
5	C61G	239				
6	2430T>C	229				
7	2201C>T	227				
8	**IVS18+66G>A**	222	1	124	3	Benign
9	IVS16-68A>G	216				
10	IVS16-92A>G	216				
11	IVS8-58delT	214				
12	**P871L**	211	1	22	7	Benign
13	**IVS7-34C>T**	207	1	124	5	Benign
14	**E1038G**	182	1	22	12	Benign
15	**K1183R**	164	1	204	16	Benign
16	R1347G	161				
17	Q356R	155				
18	4184del4	144				
19	M1008I	139				
20	R1443X	136				

Bold face indicates variants also identified in our literature search.

**Table 9.** Top 20 BIC entries for BRCA2.

	BIC designation	Count	Number of studies	Total cases (excluding controls)	Number of carriers	Pathogenicity
1	6174delT	1093				
2	**H372N**	396	1	22	13	Benign
3	10590A>C	346				
4	F599S	345				
5	IVS16-14T>C	332				
6	IVS21-66T>C	319				
7	K3326X	301				
8	I2490T	240				
9	3624A>G	234				
10	IVS11+80delTTAA	221				
11	203G>A	206				
12	**D1420Y**	200	1	102	3	Benign
13	E2856A	186				
14	7470A>G	183				
15	4035T>C	161				
16	Y42C	144				
17	S384F	143				
18	IVS8+56C>T	143				
19	P655R	142				
20	I505T	128				
	Total database entries	14 914				

Bold face indicates variants also identified in our literature search.

A search in BIC using the keyword ‘Indian’ in the ethnicity field revealed 23 BRCA1 variants and 11 BRCA2 variants. All these variants were detected in patients of Indian descent from Singapore or Malaysia. Seven of the BRCA1 variants were present in our dataset collated from the literature (Tables 10 and 11). However, of the seven variants that overlapped, only two (180delA and 185delAG) were classed as pathogenic in BIC and ClinVar (Tables 10 and 11). Of the 11 BRCA2 variants present in BIC with Indian ethnicity, three were also present in our literature dataset and of the three only one was classed as pathogenic, Q2957X. Another interesting observation was that the BRCA2 variant E1593D present in both our dataset and in the subset of 11 BIC variants, was also reported in two additional Pakistani patients in BIC.

**Table 10.** BIC searching with keyword ‘Indian’ for BRCA1.

	Exon	HGVS cDNA	HGVS Protein	Mutation	BIC Designation	BIC Class	Database	dbSNP	ClinVar Classification
BRCA1	2	**c.61_61delA**	**p.Ile21Serfs**	**F**	**180delA**	**3**	**BIC**	**-**	**Pathogenic**
	2	**c.66_67delAG**	**p.Leu22_Glu23LeuValfs**	**F**	**185delAG**	**5**	**BIC**	**rs80357713**	**Pathogenic**
	5	c.150_150delA	p.Lys50Asnfs	F	269delA	5	BIC	-	Pathogenic
	11A	c.685_685delT	p.Ser229Leufs	F	804delT	5	BIC	rs80357824	Pathogenic
	11C	c.2766_2766delA	p.Thr922 = fs	F	2885delA	5	BIC	rs80357812	Pathogenic
	11A	c.1054G>T	p.Glu352Ter	N	E352X	Class 5	BIC	rs80357472	Pathogenic
	20	c.5251C>T	p.Arg1751Ter	N	R1751X	Class 5	BIC	rs80357123	Pathogenic
	24	c.5559C>A	p.Tyr1853Ter	N	Y1853X	Pending	BIC	rs80357336	Pathogenic
	**11A**	**c.823G>A**	**p.Gly275Ser**	**M**	**G275S**	**Pending**	**BIC**	**rs8176153**	**Conflicting interpretations of pathogenicity**
	**11C**	**c.2612C>T**	**p.Pro871Leu**	**M**	**P871L**	**Class 1**	**BIC**	**rs799917**	**Benign**
	**11C**	**c.3113A>G**	**p.Glu1038Gly**	**M**	**E1038G**	**Pending**	**BIC**	**rs16941**	**Benign**
	**11D**	**c.3548A>G**	**p.Lys1183Arg**	**M**	**K1183R**	**Pending**	**BIC**	**rs16942**	**Benign**
	15	c.4643C>T	p.Thr1548Met	M	T1548M	Pending	BIC		Uncertain significance
	**16**	**c.4837A>G**	**p.Ser1613Gly**	**M**	**S1613G**	**Pending**	**BIC**	**rs1799966**	**Benign**
	5	c.135-1G>C		IVS	IVS4-1G>C	Pending	BIC		Pathogenic
	6	c.213-161A>G		IVS	IVS5-161A>G	Class 1	BIC		Benign
	9	c.548-57_548-57delT		IVS	IVS8-57delT	Pending	BIC		Benign
	13	c.4097-141A>C		IVS	IVS12-141A>C	Pending	BIC		Benign
	13	c.4186-10G>A		IVS	IVS12-10G>A	Pending	BIC	rs80358172	Conflicting interpretations of pathogenicity
	15	c.4485-90T>C		IVS	IVS14-90T>C	Pending	BIC		Uncertain significance
	15	c.4485-64C>G		IVS	IVS14-64C>G	Pending	BIC		Uncertain significance
	11B	c.2311T>C	p.Leu771 =	Syn	2430T>C	Class 1	BIC	rs16940	Benign
	**13**	**c.4308T>C**	**p.Ser1436 =**	**Syn**	**4427T>C**	**Class 1**	**BIC**	**rs1060915**	**Benign**

Bold face indicates variants also identified in our literature search

**Table 11.** BIC searching with keyword ‘Indian’ for BRCA2.

HGVS protein	Mutation	BIC designation	Clinical classification	DB	dbSNP	ClinVar classification
p.Lys1289_Cys1290?fs	F	4093del4	Class 5	BIC	rs80359412	Pathogenic
p.Gly1338_Ser1339?fs	F	4242insGG	Class 5	BIC		Pathogenic
**p.Gln2957Ter**	**N**	**Q2957X**	**Class 5**	**BIC**	**-**	**Pathogenic**
**p.Glu1593Asp**	**M**	**E1593D**	**Pending**	**BIC**	**rs80358703**	**Conflicting interpretations of pathogenicity**
p.Glu1879Lys	M	E1879K	Pending	BIC	rs55996097	Uncertain significance
**p.Ala1996Thr**	**M**	**A1996T**	**Pending**	**BIC**	**rs80358833**	**Uncertain significance**
p.Thr2310Asn	M	T2310N	Pending	BIC		Uncertain significance
p.Pro2798Leu	M	P2798L	Pending	BIC		Uncertain significance
p.Lys3115Arg	M	K3115R	Pending	BIC		Uncertain significance
p.Gln66 =	Syn	426A>G	Pending	BIC		Benign
p.Ser846 =	Syn	2766A>G	Pending	BIC		Likely benign

Bold face indicates variants also identified in our literature search.

The same search performed in ClinVar with ‘Indian’ detected 40 variants for BRCA1 and 30 for BRCA2, which included all variants also present in BIC.

Individual searches in additional databases such as TCGA, ICGC, dbSNP, GWAS catalogue, COSMIC, and HGMD did not yield any results. Although these databases contain ethnicity data, they use a very broad definition of ‘Asians’, yet the ethnicity data in the 1000genome database are region-specific and therefore this makes comparisons difficult. Furthermore, there were no data in ICGC on breast and ovarian cancers from India.

### Details of family history, penetrance, and survival in included studies

Studies in the literature used very heterogeneous criteria to define a family history of disease. Mutation prevalence in women with a family history of breast and/or ovarian cancer was presented in 11 of the 13 studies, but only seven of these provided clear criteria for family history (≥1 first degree relative affected with breast or ovarian cancer at any age). Women with sporadic breast or ovarian cancer were reported in seven publications. None of the 13 studies provided penetrance data. One small study with 91 patients presented survival information and found no significant association with pathogenic BRCA1 or BRCA2 mutations.^25^

### Histopathology

Two studies^27,28^ provided some data on breast cancer histopathology, with none describing complete histological details such as grade of cancer, hormone receptor, and HER2 status. Eachkoti *et al.* reported the majority of cases (22/25) to be infiltrating ductal carcinoma (IDC) with two inflammatory carcinomas (an aggressive type of breast cancer) and one Paget’s disease. Similarly Thirthagiri *et al.* identified IDC as the commonest histological type for both BRCA1 and BRCA2 carriers. Where grade was available, tumours were of grade 2 and 3, with no grade 1 tumours identified. BRCA1 tumours were largely triple negative and less commonly HER2 positive, whereas BRCA2 tumours were more likely to be hormone receptor positive. The data, however, were not available for the three markers in eight cases and for at least one of the three markers in an additional seven cases out of the total 28 tumours included. No studies were identified including information on the histology of ovarian tumours.

## Discussion

We have reported the findings of a methodical review of reported germline variants in BRCA1, BRCA2, and other high-penetrance breast and ovarian cancer susceptibility genes within women of Indian descent. Our searches highlight both the diversity of the Indian population as well as the paucity of data on germline variants in these genes in the Indian population. There are very limited Indian-specific data and, even where these are available, there is great variability in inclusion criteria, definition of high-risk groups (such as those with a family history), mutation detection methods, geographical origin, and ethnicity, thus making any India-wide assessment unreliable. The small cohort size mean that the spectrum of mutations identified in BRCA genes is unlikely to be representative of the Indian population and is indeterminate for other high-risk susceptibility genes in this population. Our searches have identified 18 BRCA1 and 16 BRCA2 variants in the Indian population that had not been previously reported elsewhere, nor currently present in BIC or ClinVar. There were no studies of sporadic or unselected cases and also very limited data on penetrance or survival that could be used for calculating cancer risks and hence implementing counselling and screening in Indian populations.

The spectra of BRCA1 and BRCA2 mutations have been characterized in a number of different populations worldwide, with significant variation among populations in the contributions of these genes to hereditary breast and ovarian cancer.^29^ Founder mutations account for differing proportions of cancer in different populations; for example in the Ashkenazi Jewish population [12], three founder mutations have a combined population frequency of 2% and represent 60% of breast cancer families with a BRCA1 or BRCA2 gene mutation. Similarly, BRCA1 and BRCA2 founder mutations account for 78% of families with hereditary breast cancer in Chile.^30^

Our search reveals a much lower frequency (2.3%; 39/1700) of the putative Ashkenazi founder mutation 185delAG in Indian patients with breast and/or ovarian cancer. The carriers of this mutation were usually from the south of India. Other studies have explored how this variant arose in the Indian population. Kadalmani *et al.* examined the haplotypes of carriers of this variant and their families, and concluded that it arose independently from the Ashkenazi variant. Another study by Laitman *et al.* came to a similar conclusion based on haplotype analyses of carriers from ethnically diverse backgrounds, which included Indians from Cochin, south India.^31,32^ Other founder BRCA1 and BRCA2 mutations were not detected in any of the Indian patients with breast and ovarian cancers, and no India-specific founder mutations were detected.

Our literature search shows that variation in the prevalence of high-penetrance alleles in genes such as BRCA1 and BRCA2 may contribute to the reported differences in breast and ovarian cancer incidence across India, in Indians in other countries, and between India and the west. The earlier average age of breast cancer among Indian women is especially intriguing in this respect. Data are, however, very limited and have not been collected systematically in terms of inclusion criteria, details such as family history, and critical clinical co-variates such as histopathology. Furthermore, very limited work has been published to address environmental risk factors specific to the Indian population and distinct from Western populations, such as consanguineous marriage, betel quid consumption, and pregnancies. Current guidelines on cancer screening and prevention in gene carriers are based on evidence predominantly derived from white populations of northern European origins. Work is needed to modify existing risk-prediction models such as Manchester or BOADICEA for use in women of different ethnicities. Indeed, previous work has found that overall sensitivity, specificity, and positive-predictive values were lower in the Asian population than in Caucasian populations.^26^ In conclusion, there is an urgent unmet need for large-scale studies in geographically distinct regions, with high-quality data and longitudinal studies of relatives to help elucidate the role of breast and ovarian cancer susceptibility genes in the Indian population. Understanding these differences through research to derive India-specific paradigms for diagnosis, screening, prevention, and treatment is critical and essential to improving women’s health in India.^1^ Clinics in countries with the Indian diaspora and established clinical genetics services may be able to contribute to penetrance and survival data and further tease out the differences in environmental risk factors between Indian diaspora and Indian patients.

## Supplementary Material

Supplementary Data

Supplementary Data
