# Supplementary figures and images for: Heterogeneity of germline variants in high risk breast and ovarian cancer susceptibility genes in India
Source: Precis Clin Med. 2018 Sep 22;1(2):75–87. doi: 10.1093/pcmedi/pby010 (PMC8985795; doi:10.1093/pcmedi/pby010)

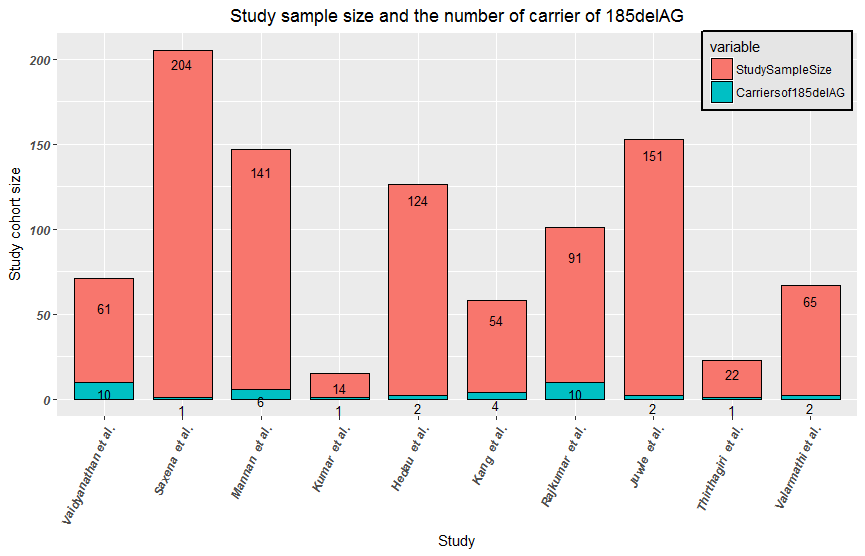


**Supplementary Figure 1.** Studies reporting the founder mutation 185delAG

Supplement: Supplementary Data [file pby010_cazier_supplementary_figure.docx]
